# Tetra-μ-benzoato-κ^4^ *O*:*O*′;κ^3^ *O*:*O*,*O*′;κ^3^ *O*,*O*′:*O*′-bis­[(benzoato-κ^2^ *O*,*O*′)(1,10-phenanthroline-κ^2^ *N*,*N*′)terbium(III)] benzoic acid disolvate

**DOI:** 10.1107/S1600536810016788

**Published:** 2010-05-15

**Authors:** Ping Howe Ooi, Siang Guan Teoh, Chin Sing Yeap, Hoong-Kun Fun

**Affiliations:** a School of Chemical Sciences, Universiti Sains Malaysia, 11800 USM, Penang, Malaysia; b X-ray Crystallography Unit, School of Physics, Universiti Sains Malaysia, 11800 USM, Penang, Malaysia

## Abstract

The asymmetric unit of the title complex, [Tb_2_(C_7_H_5_O_2_)_6_(C_12_H_8_N_2_)_2_]·2C_7_H_6_O_2_, consists of one-half of the complex mol­ecule, which lies on a crystallographic inversion centre, and one benzoic acid solvent mol­ecule. The two Tb^III^ ions are linked by four bridging benzoate ions, with a Tb⋯Tb distance of 3.9280 (6) Å. Additionally, each Tb^III^ ion is coordinated by one phenanthroline heterocycle and a bidentate benzoate ion. The irregular nine-coordinated geometry of the Tb^III^ ion is composed of seven O and two N atoms. The mol­ecular structure is stabilized by intra­molecular C—H⋯O hydrogen bonds. In the crystal structure, mol­ecules are linked into chains along the *a* axis by inter­molecular C—H⋯O hydrogen bonds. The crystal structure is further stabilized by inter­molecular C—H⋯O and C—H⋯π inter­actions. Weak π–π inter­actions are also observed [centroid–centroid distances = 3.6275 (14)–3.6604 (14) Å].

## Related literature

For general background to and applications of terbium(III) complexes, see: Xin *et al.* (2003 ▶); Tian *et al.* (2009 ▶). For related *Ln*–benzoato (*Ln* = lanthanide) complexes, see: Niu *et al.* (1999 ▶, 2002 ▶); Shi *et al.* (2001 ▶); Ooi *et al.* (2010*a* ▶,*b* ▶). For the stability of the temperature controller used for the data collection, see: Cosier & Glazer (1986 ▶).
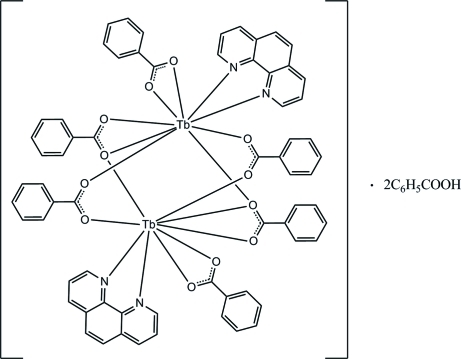


## Experimental

### 

#### Crystal data

[Tb_2_(C_7_H_5_O_2_)_6_(C_12_H_8_N_2_)_2_]·2C_7_H_6_O_2_
*M* *_r_* = 1649.14
Triclinic, 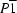

*a* = 9.5264 (15) Å
*b* = 12.719 (2) Å
*c* = 15.061 (2) Å
α = 74.836 (6)°
β = 78.345 (6)°
γ = 76.242 (6)°
*V* = 1691.7 (4) Å^3^
*Z* = 1
Mo *K*α radiation
μ = 2.15 mm^−1^
*T* = 100 K
0.59 × 0.27 × 0.13 mm

#### Data collection

Bruker APEXII DUO CCD area-detector diffractometer
Absorption correction: multi-scan (*SADABS*; Bruker, 2009 ▶) *T*_min_ = 0.364, *T*_max_ = 0.765
71560 measured reflections
11944 independent reflections
11553 reflections with *I* > 2σ(*I*)
*R*_int_ = 0.025

#### Refinement

*R*[*F* ^2^ > 2σ(*F* ^2^)] = 0.018
*wR*(*F* ^2^) = 0.101
*S* = 1.20
11944 reflections
460 parameters
H-atom parameters constrained
Δρ_max_ = 1.41 e Å^−3^
Δρ_min_ = −2.25 e Å^−3^

### 

Data collection: *APEX2* (Bruker, 2009 ▶); cell refinement: *SAINT* (Bruker, 2009 ▶); data reduction: *SAINT*; program(s) used to solve structure: *SHELXTL* (Sheldrick, 2008 ▶); program(s) used to refine structure: *SHELXTL*; molecular graphics: *SHELXTL*; software used to prepare material for publication: *SHELXTL* and *PLATON* (Spek, 2009 ▶).

## Supplementary Material

Crystal structure: contains datablocks global, I. DOI: 10.1107/S1600536810016788/rz2444sup1.cif

Structure factors: contains datablocks I. DOI: 10.1107/S1600536810016788/rz2444Isup2.hkl

Additional supplementary materials:  crystallographic information; 3D view; checkCIF report

## Figures and Tables

**Table 1:** Selected bond lengths (Å)

Tb1—O5^i^	2.3349 (14)
Tb1—O4^i^	2.3420 (15)
Tb1—O6	2.3490 (15)
Tb1—O3	2.4251 (15)
Tb1—O1	2.4669 (15)
Tb1—O2	2.4672 (15)
Tb1—N2	2.5370 (17)
Tb1—N1	2.5813 (18)
Tb1—O4	2.6057 (16)

Symmetry code: (i) 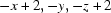.

**Table 2:** Hydrogen-bond geometry (Å, °) *Cg*1 is the centroid of the C35–C40 phenyl ring.

*D*—H⋯*A*	*D*—H	H⋯*A*	*D*⋯*A*	*D*—H⋯*A*
O8—H1*O*8⋯O1	0.82	1.88	2.640 (3)	154
C2—H2*A*⋯O5^i^	0.93	2.40	3.036 (3)	125
C4—H4*A*⋯O2^ii^	0.93	2.45	3.172 (3)	135
C11—H11*A*⋯O6	0.93	2.40	2.969 (3)	120
C15—H15*A*⋯O7	0.93	2.58	3.448 (3)	155
C23—H23*A*⋯*Cg*1^iii^	0.93	2.57	3.462 (3)	160

Symmetry codes: (i) 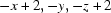; (ii) 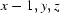; (iii) 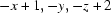.

**Fig. 1.**
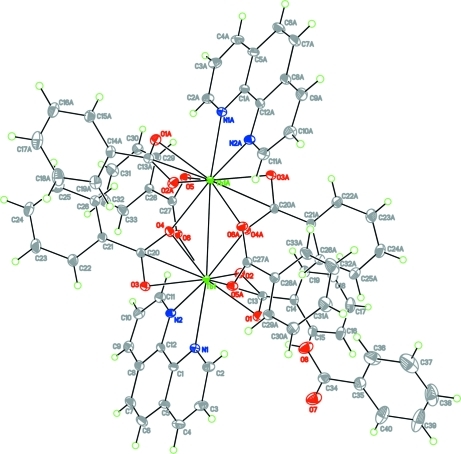
The molecular structure of the title complex, showing 20% probability displacement ellipsoids for non-H atoms and the atom-numbering scheme. The suffix A corresponds to the symmetry code [-x+2, -y, -z+2].

**Fig. 2.**
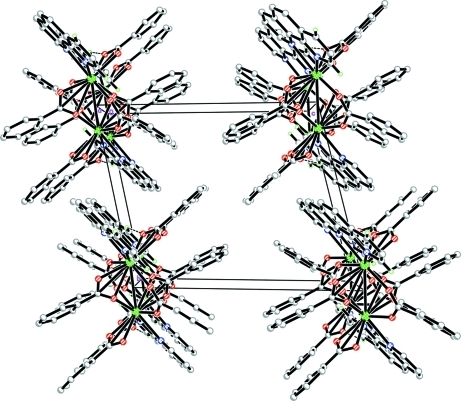
The crystal structure of the title complex, viewed along the a axis, showing four chains along a axis. The benzoic acid solvent molecules have been omitted for clarity. Intermolecular hydrogen bonds are shown as dashed lines.

## supplementary crystallographic information

### Comment

Lanthanide complexes, especially terbium and europium complexes possess excellent luminescence properties due to their narrow emission bands and are widely used in lighting devices (Xin *et al.*, 2003). Rare-earth metals with benzoic acid and some of the derivatives or with mixed ligands have drawn great attention due to the various crystal structures and unique properties (Tian *et al.*, 2009). The title compound (I) was synthesized and its structure was determined. Similar crystal structures with different lanthanides have been reported, such as lanthanum(III) (Shi *et al.*, 2001), samarium(III) (Niu *et al.*, 1999), gadolinium(III) (Niu *et al.*, 2002) and neodymium(III) (Ooi *et al.*, 2010a).

The asymmetric unit of (I) (Fig. 1) consists of one-half of the complex molecule and one benzoic acid. The complex molecule lies on a crystallographic inversion center. The geometric parameters and the configuration is very close to the related europium(III) complex (Ooi *et al.*, 2010b). The two terbium(III) ions are linked by four benzoate ions, with an Tb–Tb distance of 3.9280 (6) Å. Among the four benzoate ions, two of them also behave as chelating ligands to the terbium(III) ions. Additionally, each terbium(III) ion is coordinated by one phenanthroline heterocycle and a bidentate benzoate ion. The irregular nine-coordinated geometry of the terbium(III) ion is completed by seven benzoate O atoms and two phenanthroline N atoms. Bond lengths of Tb–O and Tb–N are listed in Table 1.

In the crystal structure, intermolecular C4—H4A···O2 hydrogen bonds (Table 2) link the molecules into chains along the *a* axis (Fig. 2). The crystal structure is further stabilized by intermolecular O8—H1O8···O1, C15A—H15A···O7 and C23—H23A···*Cg*1 interactions (Table 2; *Cg*1 is the centroid of the C35–C40 phenyl ring). Intramolecular C2—H2A···O5 and C11—H11A···O6 hydrogen bonds (Table 2) stabilize the molecular structure. Weak π–π interactions of *Cg*2···*Cg*3^iv^ = 3.6604 (14) Å and *Cg*3···*Cg*3^iv^ = 3.6275 (14) Å [*Cg*2 and *Cg*3 are centroids of rings C8–C11/N2/C12 and C1/C5–C8/C12; symmetry code: (iv) 1-x, 1-y, 2-z] are observed.

### Experimental

0.5 mmol of TbCl_3_.6H_2_O was dissolved in methanol and then was added into a solution (methanol-H_2_O, 1.5:1) of 1,10-phenanthroline (0.5 mmol) and benzoic acid (1.5 mmol). The mixture was sealed in a tube, and heated directly to 403 K. After keeping at 403 K for 2 days, it was cooled to room temperature. Colourless block crystals of the title compound were obtained by filtration, and were washed with water and ethanol.

### Refinement

All hydrogen atoms were placed in their calculated positions, with C–H = 0.93 Å, O–H = 0.82 Å, and refined using a riding model with *U*_iso_ = 1.2 *U*_eq_(C) or 1.5 *U*_eq_(O).

### Crystal data

**Table:**

[Tb_2_(C_7_H_5_O_2_)_6_(C_12_H_8_N_2_)_2_]·2C_7_H_6_O_2_	*Z* = 1
*M**_r_* = 1649.14	*F*(000) = 824
Triclinic, *P*1	*D*_x_ = 1.619 Mg m^−^^3^
Hall symbol: -P 1	Mo *K*α radiation, λ = 0.71073 Å
*a* = 9.5264 (15) Å	Cell parameters from 9691 reflections
*b* = 12.719 (2) Å	θ = 4.5–40.3°
*c* = 15.061 (2) Å	µ = 2.15 mm^−^^1^
α = 74.836 (6)°	*T* = 100 K
β = 78.345 (6)°	Block, colourless
γ = 76.242 (6)°	0.59 × 0.27 × 0.13 mm
*V* = 1691.7 (4) Å^3^	

### Data collection

**Table:**

Bruker APEXII DUO CCD area-detector diffractometer	11944 independent reflections
Radiation source: fine-focus sealed tube	11553 reflections with *I* > 2σ(*I*)
graphite	*R*_int_ = 0.025
φ and ω scans	θ_max_ = 32.5°, θ_min_ = 2.5°
Absorption correction: multi-scan (*SADABS*; Bruker, 2009)	*h* = −14→14
*T*_min_ = 0.364, *T*_max_ = 0.765	*k* = −19→19
71560 measured reflections	*l* = −22→22

### Refinement

**Table:**

Refinement on *F*^2^	Primary atom site location: structure-invariant direct methods
Least-squares matrix: full	Secondary atom site location: difference Fourier map
*R*[*F*^2^ > 2σ(*F*^2^)] = 0.018	Hydrogen site location: inferred from neighbouring sites
*wR*(*F*^2^) = 0.101	H-atom parameters constrained
*S* = 1.20	*w* = 1/[σ^2^(*F*_o_^2^) + (0.0746*P*)^2^ + 0.5014*P*] where *P* = (*F*_o_^2^ + 2*F*_c_^2^)/3
11944 reflections	(Δ/σ)_max_ = 0.006
460 parameters	Δρ_max_ = 1.41 e Å^−^^3^
0 restraints	Δρ_min_ = −2.25 e Å^−^^3^

### Special details

**Table:**

Experimental. The crystal was placed in the cold stream of an Oxford Cryosystems Cobra open-flow nitrogen cryostat (Cosier & Glazer, 1986) operating at 100.0 (1) K.
Geometry. All esds (except the esd in the dihedral angle between two l.s. planes) are estimated using the full covariance matrix. The cell esds are taken into account individually in the estimation of esds in distances, angles and torsion angles; correlations between esds in cell parameters are only used when they are defined by crystal symmetry. An approximate (isotropic) treatment of cell esds is used for estimating esds involving l.s. planes.
Refinement. Refinement of F^2^ against ALL reflections. The weighted R-factor wR and goodness of fit S are based on F^2^, conventional R-factors R are based on F, with F set to zero for negative F^2^. The threshold expression of F^2^ > 2sigma(F^2^) is used only for calculating R-factors(gt) etc. and is not relevant to the choice of reflections for refinement. R-factors based on F^2^ are statistically about twice as large as those based on F, and R- factors based on ALL data will be even larger.

### Fractional atomic coordinates and isotropic or equivalent isotropic displacement parameters (Å^2^)

**Table:**

	*x*	*y*	*z*	*U*_iso_*/*U*_eq_	
Tb1	0.854736 (8)	0.139249 (6)	0.983123 (5)	0.00821 (4)	
O1	0.81573 (17)	0.27589 (13)	0.83589 (10)	0.0136 (2)	
O2	0.99273 (17)	0.28773 (13)	0.90388 (10)	0.0142 (3)	
O3	0.71754 (17)	0.05736 (13)	1.12842 (10)	0.0151 (3)	
O4	0.92371 (16)	−0.05516 (13)	1.09240 (10)	0.0125 (2)	
O5	1.20586 (16)	−0.01538 (12)	1.08640 (10)	0.0125 (2)	
O6	1.01879 (16)	0.13125 (12)	1.08315 (10)	0.0128 (2)	
N1	0.57889 (18)	0.21253 (14)	0.97767 (12)	0.0117 (3)	
N2	0.73763 (19)	0.29920 (14)	1.06183 (12)	0.0119 (3)	
C1	0.5069 (2)	0.29323 (16)	1.02408 (13)	0.0118 (3)	
C2	0.5018 (2)	0.17375 (17)	0.93360 (15)	0.0153 (3)	
H2A	0.5502	0.1190	0.9015	0.018*	
C3	0.3507 (2)	0.21131 (19)	0.93312 (16)	0.0179 (4)	
H3A	0.3009	0.1819	0.9013	0.022*	
C4	0.2774 (2)	0.29236 (18)	0.98047 (16)	0.0172 (4)	
H4A	0.1775	0.3187	0.9808	0.021*	
C5	0.3556 (2)	0.33437 (16)	1.02809 (14)	0.0143 (3)	
C6	0.2861 (2)	0.4186 (2)	1.07950 (16)	0.0196 (4)	
H6A	0.1858	0.4447	1.0830	0.024*	
C7	0.3645 (3)	0.46038 (19)	1.12277 (16)	0.0190 (4)	
H7A	0.3176	0.5152	1.1552	0.023*	
C8	0.5192 (2)	0.42095 (17)	1.11911 (14)	0.0150 (3)	
C9	0.6044 (3)	0.46323 (18)	1.16243 (15)	0.0180 (4)	
H9A	0.5608	0.5164	1.1970	0.022*	
C10	0.7530 (3)	0.42479 (18)	1.15290 (15)	0.0184 (4)	
H10A	0.8116	0.4523	1.1802	0.022*	
C11	0.8150 (3)	0.34356 (19)	1.10146 (15)	0.0160 (4)	
H11A	0.9159	0.3193	1.0946	0.019*	
C12	0.5909 (2)	0.33772 (16)	1.06979 (13)	0.0120 (3)	
C13	0.9288 (2)	0.31535 (16)	0.83381 (13)	0.0117 (3)	
C14	0.9857 (2)	0.39245 (16)	0.74819 (13)	0.0132 (3)	
C15	0.8946 (2)	0.45653 (17)	0.68380 (14)	0.0154 (3)	
H15A	0.7970	0.4505	0.6929	0.019*	
C16	0.9508 (3)	0.52973 (19)	0.60562 (15)	0.0204 (4)	
H16A	0.8900	0.5739	0.5633	0.024*	
C17	1.0978 (3)	0.5369 (2)	0.59085 (16)	0.0234 (4)	
H17A	1.1347	0.5859	0.5387	0.028*	
C18	1.1888 (3)	0.4715 (2)	0.65327 (16)	0.0222 (4)	
H18A	1.2873	0.4753	0.6425	0.027*	
C19	1.1326 (2)	0.39948 (18)	0.73292 (15)	0.0165 (3)	
H19A	1.1934	0.3563	0.7756	0.020*	
C20	0.8169 (2)	−0.02502 (16)	1.15246 (13)	0.0113 (3)	
C21	0.8118 (2)	−0.08378 (16)	1.25201 (13)	0.0119 (3)	
C22	0.6784 (2)	−0.0938 (2)	1.30829 (15)	0.0180 (4)	
H22A	0.5919	−0.0662	1.2831	0.022*	
C23	0.6744 (3)	−0.1448 (2)	1.40201 (16)	0.0234 (4)	
H23A	0.5852	−0.1519	1.4396	0.028*	
C24	0.8026 (3)	−0.1851 (2)	1.43949 (16)	0.0232 (4)	
H24A	0.7993	−0.2187	1.5024	0.028*	
C25	0.9368 (3)	−0.1757 (2)	1.38402 (15)	0.0211 (4)	
H25A	1.0230	−0.2035	1.4096	0.025*	
C26	0.9411 (2)	−0.12441 (18)	1.28979 (14)	0.0156 (3)	
H26A	1.0303	−0.1174	1.2523	0.019*	
C27	1.1338 (2)	0.07212 (17)	1.11195 (13)	0.0114 (3)	
C28	1.1887 (2)	0.11019 (17)	1.18255 (13)	0.0123 (3)	
C29	1.3377 (2)	0.09517 (17)	1.18401 (13)	0.0133 (3)	
H29A	1.4044	0.0546	1.1451	0.016*	
C30	1.3869 (2)	0.1408 (2)	1.24357 (15)	0.0178 (4)	
H30A	1.4866	0.1326	1.2431	0.021*	
C31	1.2878 (3)	0.1983 (2)	1.30368 (18)	0.0250 (5)	
H31A	1.3213	0.2286	1.3435	0.030*	
C32	1.1377 (3)	0.2110 (3)	1.30473 (19)	0.0289 (5)	
H32A	1.0712	0.2481	1.3462	0.035*	
C33	1.0880 (2)	0.1681 (2)	1.24358 (16)	0.0198 (4)	
H33A	0.9883	0.1778	1.2431	0.024*	
O7	0.5748 (2)	0.39406 (17)	0.65710 (15)	0.0275 (4)	
O8	0.7119 (2)	0.22746 (16)	0.70482 (13)	0.0232 (3)	
H1O8	0.7180	0.2548	0.7472	0.035*	
C34	0.6364 (2)	0.3025 (2)	0.64473 (16)	0.0182 (4)	
C35	0.6377 (3)	0.2640 (2)	0.55910 (16)	0.0204 (4)	
C36	0.7126 (3)	0.1611 (3)	0.5463 (2)	0.0314 (6)	
H36A	0.7601	0.1113	0.5933	0.038*	
C37	0.7172 (4)	0.1314 (4)	0.4626 (3)	0.0471 (10)	
H37A	0.7685	0.0621	0.4536	0.056*	
C38	0.6459 (4)	0.2047 (4)	0.3932 (2)	0.0448 (9)	
H38A	0.6503	0.1851	0.3373	0.054*	
C39	0.5691 (4)	0.3057 (3)	0.40607 (19)	0.0409 (9)	
H39A	0.5201	0.3541	0.3592	0.049*	
C40	0.5630 (4)	0.3373 (3)	0.48907 (18)	0.0290 (5)	
H40B	0.5098	0.4063	0.4977	0.035*	

### Atomic displacement parameters (Å^2^)

**Table:**

	*U*^11^	*U*^22^	*U*^33^	*U*^12^	*U*^13^	*U*^23^
Tb1	0.00733 (6)	0.00899 (6)	0.00856 (6)	−0.00089 (3)	−0.00166 (3)	−0.00267 (3)
O1	0.0125 (6)	0.0153 (6)	0.0137 (6)	−0.0053 (5)	−0.0031 (5)	−0.0015 (5)
O2	0.0145 (6)	0.0148 (6)	0.0135 (6)	−0.0041 (5)	−0.0041 (5)	−0.0012 (5)
O3	0.0120 (6)	0.0155 (6)	0.0137 (6)	0.0016 (5)	−0.0005 (5)	−0.0009 (5)
O4	0.0110 (6)	0.0155 (6)	0.0114 (5)	−0.0027 (5)	0.0005 (4)	−0.0052 (5)
O5	0.0111 (6)	0.0112 (6)	0.0162 (6)	−0.0001 (5)	−0.0036 (5)	−0.0056 (5)
O6	0.0123 (6)	0.0129 (6)	0.0140 (6)	0.0006 (5)	−0.0055 (5)	−0.0047 (5)
N1	0.0100 (6)	0.0117 (6)	0.0133 (6)	−0.0016 (5)	−0.0019 (5)	−0.0033 (5)
N2	0.0117 (7)	0.0103 (6)	0.0144 (7)	−0.0006 (5)	−0.0039 (5)	−0.0038 (5)
C1	0.0095 (7)	0.0115 (7)	0.0132 (7)	−0.0010 (6)	−0.0017 (6)	−0.0016 (6)
C2	0.0132 (8)	0.0156 (8)	0.0190 (8)	−0.0026 (7)	−0.0056 (7)	−0.0049 (6)
C3	0.0131 (8)	0.0191 (9)	0.0231 (9)	−0.0046 (7)	−0.0076 (7)	−0.0024 (7)
C4	0.0103 (8)	0.0156 (8)	0.0230 (9)	−0.0016 (7)	−0.0034 (7)	0.0001 (7)
C5	0.0098 (7)	0.0115 (7)	0.0182 (8)	0.0005 (6)	−0.0011 (6)	−0.0007 (6)
C6	0.0123 (8)	0.0211 (9)	0.0208 (9)	−0.0001 (7)	0.0028 (7)	−0.0034 (7)
C7	0.0172 (9)	0.0174 (9)	0.0183 (9)	0.0024 (7)	0.0025 (7)	−0.0059 (7)
C8	0.0160 (8)	0.0123 (8)	0.0141 (7)	0.0011 (6)	−0.0003 (6)	−0.0037 (6)
C9	0.0240 (10)	0.0142 (8)	0.0153 (8)	0.0009 (7)	−0.0027 (7)	−0.0071 (6)
C10	0.0236 (10)	0.0154 (8)	0.0187 (9)	−0.0010 (7)	−0.0066 (7)	−0.0082 (7)
C11	0.0162 (9)	0.0178 (9)	0.0173 (9)	−0.0042 (7)	−0.0051 (7)	−0.0070 (7)
C12	0.0114 (7)	0.0127 (7)	0.0113 (7)	−0.0019 (6)	−0.0001 (6)	−0.0032 (6)
C13	0.0114 (8)	0.0116 (7)	0.0126 (7)	−0.0021 (6)	−0.0021 (6)	−0.0032 (6)
C14	0.0159 (8)	0.0128 (7)	0.0111 (7)	−0.0048 (6)	−0.0003 (6)	−0.0028 (6)
C15	0.0190 (9)	0.0139 (8)	0.0127 (7)	−0.0019 (7)	−0.0023 (6)	−0.0031 (6)
C16	0.0290 (11)	0.0172 (9)	0.0133 (8)	−0.0050 (8)	−0.0025 (7)	−0.0007 (7)
C17	0.0335 (12)	0.0222 (10)	0.0147 (8)	−0.0130 (9)	0.0014 (8)	−0.0018 (7)
C18	0.0248 (11)	0.0269 (11)	0.0177 (9)	−0.0154 (9)	0.0022 (8)	−0.0046 (8)
C19	0.0173 (9)	0.0181 (9)	0.0159 (8)	−0.0072 (7)	−0.0013 (7)	−0.0046 (7)
C20	0.0098 (7)	0.0130 (7)	0.0110 (7)	−0.0032 (6)	−0.0006 (6)	−0.0024 (6)
C21	0.0120 (7)	0.0111 (7)	0.0113 (7)	−0.0016 (6)	−0.0006 (6)	−0.0017 (5)
C22	0.0144 (9)	0.0232 (10)	0.0148 (8)	−0.0069 (7)	−0.0003 (7)	0.0002 (7)
C23	0.0208 (10)	0.0307 (12)	0.0154 (9)	−0.0106 (9)	0.0016 (7)	0.0021 (8)
C24	0.0264 (11)	0.0269 (11)	0.0129 (8)	−0.0065 (9)	−0.0020 (8)	0.0020 (7)
C25	0.0204 (10)	0.0266 (11)	0.0147 (8)	−0.0022 (8)	−0.0061 (7)	−0.0014 (7)
C26	0.0146 (8)	0.0183 (9)	0.0133 (8)	−0.0021 (7)	−0.0025 (6)	−0.0033 (6)
C27	0.0111 (8)	0.0137 (8)	0.0107 (7)	−0.0038 (6)	−0.0019 (6)	−0.0037 (6)
C28	0.0118 (7)	0.0151 (8)	0.0118 (7)	−0.0025 (6)	−0.0027 (6)	−0.0055 (6)
C29	0.0122 (8)	0.0151 (8)	0.0141 (7)	−0.0018 (6)	−0.0033 (6)	−0.0053 (6)
C30	0.0156 (9)	0.0245 (10)	0.0174 (8)	−0.0053 (8)	−0.0049 (7)	−0.0086 (7)
C31	0.0202 (10)	0.0392 (13)	0.0236 (10)	−0.0056 (9)	−0.0043 (8)	−0.0201 (10)
C32	0.0206 (10)	0.0470 (16)	0.0279 (11)	−0.0046 (10)	−0.0008 (9)	−0.0278 (11)
C33	0.0130 (8)	0.0307 (11)	0.0194 (9)	−0.0010 (8)	−0.0019 (7)	−0.0149 (8)
O7	0.0284 (9)	0.0259 (9)	0.0323 (9)	0.0025 (7)	−0.0143 (8)	−0.0129 (7)
O8	0.0244 (8)	0.0232 (8)	0.0253 (8)	0.0009 (7)	−0.0132 (7)	−0.0091 (6)
C34	0.0146 (8)	0.0219 (9)	0.0198 (9)	−0.0035 (7)	−0.0049 (7)	−0.0061 (7)
C35	0.0173 (9)	0.0289 (11)	0.0193 (9)	−0.0096 (8)	−0.0028 (7)	−0.0084 (8)
C36	0.0209 (11)	0.0417 (15)	0.0413 (15)	−0.0014 (10)	−0.0090 (10)	−0.0269 (12)
C37	0.0299 (14)	0.073 (3)	0.057 (2)	−0.0113 (16)	−0.0017 (14)	−0.050 (2)
C38	0.0393 (17)	0.082 (3)	0.0297 (14)	−0.0327 (19)	0.0044 (12)	−0.0306 (16)
C39	0.057 (2)	0.061 (2)	0.0171 (10)	−0.0429 (19)	−0.0069 (11)	−0.0013 (11)
C40	0.0395 (14)	0.0340 (13)	0.0197 (10)	−0.0232 (12)	−0.0092 (9)	0.0011 (9)

### Geometric parameters (Å, °)

**Table:**

Tb1—O5^i^	2.3349 (14)	C15—H15A	0.9300
Tb1—O4^i^	2.3420 (15)	C16—C17	1.394 (4)
Tb1—O6	2.3490 (15)	C16—H16A	0.9300
Tb1—O3	2.4251 (15)	C17—C18	1.380 (4)
Tb1—O1	2.4669 (15)	C17—H17A	0.9300
Tb1—O2	2.4672 (15)	C18—C19	1.402 (3)
Tb1—N2	2.5370 (17)	C18—H18A	0.9300
Tb1—N1	2.5813 (18)	C19—H19A	0.9300
Tb1—O4	2.6057 (16)	C20—C21	1.487 (3)
Tb1—C13	2.8367 (19)	C21—C22	1.391 (3)
Tb1—C20	2.8633 (19)	C21—C26	1.391 (3)
Tb1—Tb1^i^	3.9280 (6)	C22—C23	1.388 (3)
O1—C13	1.284 (2)	C22—H22A	0.9300
O2—C13	1.254 (2)	C23—C24	1.379 (4)
O3—C20	1.262 (3)	C23—H23A	0.9300
O4—C20	1.274 (2)	C24—C25	1.391 (3)
O4—Tb1^i^	2.3420 (15)	C24—H24A	0.9300
O5—C27	1.269 (2)	C25—C26	1.395 (3)
O5—Tb1^i^	2.3348 (14)	C25—H25A	0.9300
O6—C27	1.259 (2)	C26—H26A	0.9300
N1—C2	1.328 (3)	C27—C28	1.501 (3)
N1—C1	1.364 (2)	C28—C29	1.390 (3)
N2—C11	1.330 (3)	C28—C33	1.404 (3)
N2—C12	1.358 (3)	C29—C30	1.390 (3)
C1—C5	1.406 (3)	C29—H29A	0.9300
C1—C12	1.441 (3)	C30—C31	1.385 (3)
C2—C3	1.406 (3)	C30—H30A	0.9300
C2—H2A	0.9300	C31—C32	1.398 (4)
C3—C4	1.378 (3)	C31—H31A	0.9300
C3—H3A	0.9300	C32—C33	1.389 (3)
C4—C5	1.401 (3)	C32—H32A	0.9300
C4—H4A	0.9300	C33—H33A	0.9300
C5—C6	1.440 (3)	O7—C34	1.217 (3)
C6—C7	1.353 (4)	O8—C34	1.316 (3)
C6—H6A	0.9300	O8—H1O8	0.8200
C7—C8	1.434 (3)	C34—C35	1.491 (3)
C7—H7A	0.9300	C35—C36	1.376 (4)
C8—C9	1.407 (3)	C35—C40	1.400 (4)
C8—C12	1.415 (3)	C36—C37	1.397 (4)
C9—C10	1.375 (3)	C36—H36A	0.9300
C9—H9A	0.9300	C37—C38	1.378 (7)
C10—C11	1.400 (3)	C37—H37A	0.9300
C10—H10A	0.9300	C38—C39	1.359 (6)
C11—H11A	0.9300	C38—H38A	0.9300
C13—C14	1.493 (3)	C39—C40	1.397 (4)
C14—C19	1.391 (3)	C39—H39A	0.9300
C14—C15	1.396 (3)	C40—H40B	0.9300
C15—C16	1.396 (3)		
			
O5^i^—Tb1—O4^i^	74.50 (5)	C10—C9—C8	119.06 (19)
O5^i^—Tb1—O6	136.68 (5)	C10—C9—H9A	120.5
O4^i^—Tb1—O6	78.07 (5)	C8—C9—H9A	120.5
O5^i^—Tb1—O3	88.57 (5)	C9—C10—C11	119.1 (2)
O4^i^—Tb1—O3	126.68 (5)	C9—C10—H10A	120.5
O6—Tb1—O3	81.63 (5)	C11—C10—H10A	120.5
O5^i^—Tb1—O1	85.28 (5)	N2—C11—C10	123.6 (2)
O4^i^—Tb1—O1	89.11 (5)	N2—C11—H11A	118.2
O6—Tb1—O1	127.31 (5)	C10—C11—H11A	118.2
O3—Tb1—O1	140.33 (5)	N2—C12—C8	122.55 (18)
O5^i^—Tb1—O2	126.18 (5)	N2—C12—C1	118.12 (17)
O4^i^—Tb1—O2	73.03 (5)	C8—C12—C1	119.32 (18)
O6—Tb1—O2	74.65 (5)	O2—C13—O1	119.67 (18)
O3—Tb1—O2	145.10 (5)	O2—C13—C14	119.87 (18)
O1—Tb1—O2	52.80 (5)	O1—C13—C14	120.46 (18)
O5^i^—Tb1—N2	141.13 (5)	O2—C13—Tb1	60.23 (10)
O4^i^—Tb1—N2	143.06 (5)	O1—C13—Tb1	60.29 (10)
O6—Tb1—N2	76.18 (5)	C14—C13—Tb1	170.03 (14)
O3—Tb1—N2	74.81 (5)	C19—C14—C15	120.02 (18)
O1—Tb1—N2	85.87 (5)	C19—C14—C13	118.85 (18)
O2—Tb1—N2	74.91 (5)	C15—C14—C13	121.13 (18)
O5^i^—Tb1—N1	77.20 (5)	C16—C15—C14	119.5 (2)
O4^i^—Tb1—N1	145.78 (5)	C16—C15—H15A	120.2
O6—Tb1—N1	136.14 (5)	C14—C15—H15A	120.2
O3—Tb1—N1	70.78 (5)	C17—C16—C15	120.2 (2)
O1—Tb1—N1	69.64 (5)	C17—C16—H16A	119.9
O2—Tb1—N1	110.26 (5)	C15—C16—H16A	119.9
N2—Tb1—N1	64.24 (5)	C18—C17—C16	120.2 (2)
O5^i^—Tb1—O4	75.20 (5)	C18—C17—H17A	119.9
O4^i^—Tb1—O4	75.02 (5)	C16—C17—H17A	119.9
O6—Tb1—O4	65.64 (5)	C17—C18—C19	119.9 (2)
O3—Tb1—O4	51.71 (5)	C17—C18—H18A	120.0
O1—Tb1—O4	157.45 (5)	C19—C18—H18A	120.0
O2—Tb1—O4	133.00 (5)	C14—C19—C18	120.0 (2)
N2—Tb1—O4	116.44 (5)	C14—C19—H19A	120.0
N1—Tb1—O4	115.60 (5)	C18—C19—H19A	120.0
O5^i^—Tb1—C13	105.11 (6)	O3—C20—O4	120.25 (18)
O4^i^—Tb1—C13	77.50 (6)	O3—C20—C21	119.52 (17)
O6—Tb1—C13	100.82 (6)	O4—C20—C21	120.18 (18)
O3—Tb1—C13	155.23 (5)	O3—C20—Tb1	57.25 (10)
O1—Tb1—C13	26.87 (5)	O4—C20—Tb1	65.44 (10)
O2—Tb1—C13	26.18 (5)	C21—C20—Tb1	161.59 (13)
N2—Tb1—C13	81.81 (6)	C22—C21—C26	119.98 (18)
N1—Tb1—C13	91.87 (6)	C22—C21—C20	120.43 (18)
O4—Tb1—C13	151.34 (5)	C26—C21—C20	119.53 (17)
O5^i^—Tb1—C20	85.27 (6)	C23—C22—C21	120.0 (2)
O4^i^—Tb1—C20	101.22 (6)	C23—C22—H22A	120.0
O6—Tb1—C20	67.92 (5)	C21—C22—H22A	120.0
O3—Tb1—C20	25.96 (5)	C24—C23—C22	120.0 (2)
O1—Tb1—C20	163.59 (5)	C24—C23—H23A	120.0
O2—Tb1—C20	142.47 (5)	C22—C23—H23A	120.0
N2—Tb1—C20	93.14 (6)	C23—C24—C25	120.6 (2)
N1—Tb1—C20	95.17 (5)	C23—C24—H24A	119.7
O4—Tb1—C20	26.41 (5)	C25—C24—H24A	119.7
C13—Tb1—C20	168.56 (6)	C24—C25—C26	119.5 (2)
O5^i^—Tb1—Tb1^i^	70.80 (4)	C24—C25—H25A	120.3
O4^i^—Tb1—Tb1^i^	39.85 (4)	C26—C25—H25A	120.3
O6—Tb1—Tb1^i^	66.62 (4)	C21—C26—C25	119.9 (2)
O3—Tb1—Tb1^i^	86.85 (4)	C21—C26—H26A	120.0
O1—Tb1—Tb1^i^	127.12 (4)	C25—C26—H26A	120.0
O2—Tb1—Tb1^i^	106.16 (4)	O6—C27—O5	125.75 (18)
N2—Tb1—Tb1^i^	140.52 (4)	O6—C27—C28	116.05 (18)
N1—Tb1—Tb1^i^	141.23 (4)	O5—C27—C28	118.20 (18)
O4—Tb1—Tb1^i^	35.17 (3)	C29—C28—C33	119.88 (18)
C13—Tb1—Tb1^i^	116.94 (4)	C29—C28—C27	120.82 (17)
C20—Tb1—Tb1^i^	61.44 (4)	C33—C28—C27	119.16 (18)
C13—O1—Tb1	92.84 (12)	C30—C29—C28	120.00 (19)
C13—O2—Tb1	93.60 (12)	C30—C29—H29A	120.0
C20—O3—Tb1	96.79 (12)	C28—C29—H29A	120.0
C20—O4—Tb1^i^	164.95 (14)	C31—C30—C29	120.2 (2)
C20—O4—Tb1	88.14 (12)	C31—C30—H30A	119.9
Tb1^i^—O4—Tb1	104.98 (5)	C29—C30—H30A	119.9
C27—O5—Tb1^i^	132.94 (13)	C30—C31—C32	120.2 (2)
C27—O6—Tb1	141.05 (13)	C30—C31—H31A	119.9
C2—N1—C1	117.85 (18)	C32—C31—H31A	119.9
C2—N1—Tb1	123.32 (14)	C33—C32—C31	119.8 (2)
C1—N1—Tb1	118.81 (12)	C33—C32—H32A	120.1
C11—N2—C12	117.72 (18)	C31—C32—H32A	120.1
C11—N2—Tb1	121.72 (14)	C32—C33—C28	119.9 (2)
C12—N2—Tb1	120.44 (12)	C32—C33—H33A	120.1
N1—C1—C5	122.49 (18)	C28—C33—H33A	120.1
N1—C1—C12	118.00 (17)	C34—O8—H1O8	109.5
C5—C1—C12	119.50 (18)	O7—C34—O8	123.5 (2)
N1—C2—C3	123.3 (2)	O7—C34—C35	123.8 (2)
N1—C2—H2A	118.4	O8—C34—C35	112.7 (2)
C3—C2—H2A	118.4	C36—C35—C40	119.7 (2)
C4—C3—C2	119.08 (19)	C36—C35—C34	122.1 (2)
C4—C3—H3A	120.5	C40—C35—C34	118.2 (2)
C2—C3—H3A	120.5	C35—C36—C37	119.9 (3)
C3—C4—C5	118.96 (19)	C35—C36—H36A	120.1
C3—C4—H4A	120.5	C37—C36—H36A	120.1
C5—C4—H4A	120.5	C38—C37—C36	120.1 (4)
C4—C5—C1	118.35 (19)	C38—C37—H37A	120.0
C4—C5—C6	122.03 (19)	C36—C37—H37A	120.0
C1—C5—C6	119.62 (19)	C39—C38—C37	120.4 (3)
C7—C6—C5	121.1 (2)	C39—C38—H38A	119.8
C7—C6—H6A	119.5	C37—C38—H38A	119.8
C5—C6—H6A	119.5	C38—C39—C40	120.6 (3)
C6—C7—C8	120.6 (2)	C38—C39—H39A	119.7
C6—C7—H7A	119.7	C40—C39—H39A	119.7
C8—C7—H7A	119.7	C39—C40—C35	119.3 (3)
C9—C8—C12	117.9 (2)	C39—C40—H40B	120.4
C9—C8—C7	122.18 (19)	C35—C40—H40B	120.4
C12—C8—C7	119.9 (2)		
			
O5^i^—Tb1—O1—C13	−137.88 (12)	C9—C10—C11—N2	−1.2 (3)
O4^i^—Tb1—O1—C13	−63.36 (12)	C11—N2—C12—C8	−0.9 (3)
O6—Tb1—O1—C13	10.87 (14)	Tb1—N2—C12—C8	175.14 (14)
O3—Tb1—O1—C13	140.10 (12)	C11—N2—C12—C1	177.66 (18)
O2—Tb1—O1—C13	5.85 (11)	Tb1—N2—C12—C1	−6.3 (2)
N2—Tb1—O1—C13	80.01 (12)	C9—C8—C12—N2	−1.1 (3)
N1—Tb1—O1—C13	144.03 (13)	C7—C8—C12—N2	178.23 (19)
O4—Tb1—O1—C13	−108.03 (15)	C9—C8—C12—C1	−179.56 (18)
C20—Tb1—O1—C13	167.10 (17)	C7—C8—C12—C1	−0.3 (3)
Tb1^i^—Tb1—O1—C13	−76.32 (12)	N1—C1—C12—N2	1.9 (3)
O5^i^—Tb1—O2—C13	40.92 (14)	C5—C1—C12—N2	−177.64 (18)
O4^i^—Tb1—O2—C13	96.20 (13)	N1—C1—C12—C8	−179.53 (18)
O6—Tb1—O2—C13	178.13 (13)	C5—C1—C12—C8	0.9 (3)
O3—Tb1—O2—C13	−132.94 (12)	Tb1—O2—C13—O1	10.6 (2)
O1—Tb1—O2—C13	−6.00 (11)	Tb1—O2—C13—C14	−168.50 (16)
N2—Tb1—O2—C13	−102.38 (13)	Tb1—O1—C13—O2	−10.6 (2)
N1—Tb1—O2—C13	−47.79 (13)	Tb1—O1—C13—C14	168.50 (16)
O4—Tb1—O2—C13	145.35 (11)	O5^i^—Tb1—C13—O2	−146.80 (12)
C20—Tb1—O2—C13	−177.43 (11)	O4^i^—Tb1—C13—O2	−76.88 (12)
Tb1^i^—Tb1—O2—C13	118.66 (12)	O6—Tb1—C13—O2	−1.83 (13)
O5^i^—Tb1—O3—C20	82.13 (13)	O3—Tb1—C13—O2	91.64 (17)
O4^i^—Tb1—O3—C20	12.74 (15)	O1—Tb1—C13—O2	169.4 (2)
O6—Tb1—O3—C20	−55.54 (12)	N2—Tb1—C13—O2	72.32 (12)
O1—Tb1—O3—C20	162.97 (11)	N1—Tb1—C13—O2	135.96 (12)
O2—Tb1—O3—C20	−102.83 (14)	O4—Tb1—C13—O2	−60.11 (17)
N2—Tb1—O3—C20	−133.41 (13)	C20—Tb1—C13—O2	7.9 (3)
N1—Tb1—O3—C20	159.07 (14)	Tb1^i^—Tb1—C13—O2	−70.97 (12)
O4—Tb1—O3—C20	9.94 (11)	O5^i^—Tb1—C13—O1	43.82 (12)
C13—Tb1—O3—C20	−153.24 (15)	O4^i^—Tb1—C13—O1	113.73 (12)
Tb1^i^—Tb1—O3—C20	11.28 (12)	O6—Tb1—C13—O1	−171.22 (11)
O5^i^—Tb1—O4—C20	−109.90 (12)	O3—Tb1—C13—O1	−77.75 (18)
O4^i^—Tb1—O4—C20	172.54 (14)	O2—Tb1—C13—O1	−169.4 (2)
O6—Tb1—O4—C20	89.07 (12)	N2—Tb1—C13—O1	−97.07 (12)
O3—Tb1—O4—C20	−9.78 (11)	N1—Tb1—C13—O1	−33.43 (12)
O1—Tb1—O4—C20	−140.77 (14)	O4—Tb1—C13—O1	130.51 (12)
O2—Tb1—O4—C20	124.05 (11)	C20—Tb1—C13—O1	−161.5 (2)
N2—Tb1—O4—C20	30.26 (13)	Tb1^i^—Tb1—C13—O1	119.65 (11)
N1—Tb1—O4—C20	−42.28 (12)	O2—C13—C14—C19	24.2 (3)
C13—Tb1—O4—C20	155.58 (14)	O1—C13—C14—C19	−154.9 (2)
Tb1^i^—Tb1—O4—C20	172.54 (14)	O2—C13—C14—C15	−156.1 (2)
O5^i^—Tb1—O4—Tb1^i^	77.56 (6)	O1—C13—C14—C15	24.8 (3)
O4^i^—Tb1—O4—Tb1^i^	0.0	C19—C14—C15—C16	−1.8 (3)
O6—Tb1—O4—Tb1^i^	−83.46 (6)	C13—C14—C15—C16	178.46 (19)
O3—Tb1—O4—Tb1^i^	177.68 (9)	C14—C15—C16—C17	1.5 (3)
O1—Tb1—O4—Tb1^i^	46.69 (15)	C15—C16—C17—C18	0.1 (4)
O2—Tb1—O4—Tb1^i^	−48.49 (8)	C16—C17—C18—C19	−1.4 (4)
N2—Tb1—O4—Tb1^i^	−142.27 (5)	C15—C14—C19—C18	0.5 (3)
N1—Tb1—O4—Tb1^i^	145.18 (5)	C13—C14—C19—C18	−179.75 (19)
C13—Tb1—O4—Tb1^i^	−16.96 (13)	C17—C18—C19—C14	1.1 (3)
C20—Tb1—O4—Tb1^i^	−172.54 (14)	Tb1—O3—C20—O4	−18.7 (2)
O5^i^—Tb1—O6—C27	19.0 (2)	Tb1—O3—C20—C21	158.78 (15)
O4^i^—Tb1—O6—C27	−32.5 (2)	Tb1^i^—O4—C20—O3	168.3 (4)
O3—Tb1—O6—C27	97.9 (2)	Tb1—O4—C20—O3	17.25 (19)
O1—Tb1—O6—C27	−112.1 (2)	Tb1^i^—O4—C20—C21	−9.1 (6)
O2—Tb1—O6—C27	−107.9 (2)	Tb1—O4—C20—C21	−160.22 (16)
N2—Tb1—O6—C27	174.2 (2)	Tb1^i^—O4—C20—Tb1	151.1 (5)
N1—Tb1—O6—C27	148.63 (19)	O5^i^—Tb1—C20—O3	−96.46 (13)
O4—Tb1—O6—C27	46.3 (2)	O4^i^—Tb1—C20—O3	−169.61 (12)
C13—Tb1—O6—C27	−107.1 (2)	O6—Tb1—C20—O3	118.32 (13)
C20—Tb1—O6—C27	75.0 (2)	O1—Tb1—C20—O3	−41.4 (3)
Tb1^i^—Tb1—O6—C27	7.7 (2)	O2—Tb1—C20—O3	113.70 (13)
O5^i^—Tb1—N1—C2	−7.84 (15)	N2—Tb1—C20—O3	44.60 (13)
O4^i^—Tb1—N1—C2	26.9 (2)	N1—Tb1—C20—O3	−19.80 (13)
O6—Tb1—N1—C2	−155.01 (14)	O4—Tb1—C20—O3	−162.27 (19)
O3—Tb1—N1—C2	−100.83 (16)	C13—Tb1—C20—O3	108.0 (3)
O1—Tb1—N1—C2	81.83 (16)	Tb1^i^—Tb1—C20—O3	−167.15 (14)
O2—Tb1—N1—C2	116.32 (16)	O5^i^—Tb1—C20—O4	65.81 (11)
N2—Tb1—N1—C2	177.22 (17)	O4^i^—Tb1—C20—O4	−7.35 (14)
O4—Tb1—N1—C2	−74.30 (16)	O6—Tb1—C20—O4	−79.41 (11)
C13—Tb1—N1—C2	97.24 (16)	O3—Tb1—C20—O4	162.27 (19)
C20—Tb1—N1—C2	−91.79 (16)	O1—Tb1—C20—O4	120.83 (19)
Tb1^i^—Tb1—N1—C2	−42.63 (18)	O2—Tb1—C20—O4	−84.03 (14)
O5^i^—Tb1—N1—C1	170.48 (14)	N2—Tb1—C20—O4	−153.13 (11)
O4^i^—Tb1—N1—C1	−154.75 (12)	N1—Tb1—C20—O4	142.47 (11)
O6—Tb1—N1—C1	23.32 (17)	C13—Tb1—C20—O4	−89.8 (3)
O3—Tb1—N1—C1	77.50 (14)	Tb1^i^—Tb1—C20—O4	−4.89 (9)
O1—Tb1—N1—C1	−99.84 (14)	O5^i^—Tb1—C20—C21	178.0 (4)
O2—Tb1—N1—C1	−65.36 (14)	O4^i^—Tb1—C20—C21	104.8 (4)
N2—Tb1—N1—C1	−4.46 (13)	O6—Tb1—C20—C21	32.8 (4)
O4—Tb1—N1—C1	104.02 (14)	O3—Tb1—C20—C21	−85.6 (4)
C13—Tb1—N1—C1	−84.44 (14)	O1—Tb1—C20—C21	−127.0 (4)
C20—Tb1—N1—C1	86.54 (14)	O2—Tb1—C20—C21	28.1 (5)
Tb1^i^—Tb1—N1—C1	135.70 (12)	N2—Tb1—C20—C21	−41.0 (4)
O5^i^—Tb1—N2—C11	173.53 (14)	N1—Tb1—C20—C21	−105.4 (4)
O4^i^—Tb1—N2—C11	−26.2 (2)	O4—Tb1—C20—C21	112.2 (5)
O6—Tb1—N2—C11	20.83 (16)	C13—Tb1—C20—C21	22.4 (6)
O3—Tb1—N2—C11	105.76 (16)	Tb1^i^—Tb1—C20—C21	107.3 (4)
O1—Tb1—N2—C11	−109.23 (16)	O3—C20—C21—C22	34.3 (3)
O2—Tb1—N2—C11	−56.70 (16)	O4—C20—C21—C22	−148.2 (2)
N1—Tb1—N2—C11	−178.59 (18)	Tb1—C20—C21—C22	108.8 (4)
O4—Tb1—N2—C11	74.21 (17)	O3—C20—C21—C26	−142.7 (2)
C13—Tb1—N2—C11	−82.50 (16)	O4—C20—C21—C26	34.8 (3)
C20—Tb1—N2—C11	87.18 (16)	Tb1—C20—C21—C26	−68.2 (5)
Tb1^i^—Tb1—N2—C11	40.54 (19)	C26—C21—C22—C23	−0.4 (3)
O5^i^—Tb1—N2—C12	−2.31 (19)	C20—C21—C22—C23	−177.4 (2)
O4^i^—Tb1—N2—C12	157.94 (13)	C21—C22—C23—C24	0.4 (4)
O6—Tb1—N2—C12	−155.01 (15)	C22—C23—C24—C25	−0.4 (4)
O3—Tb1—N2—C12	−70.09 (14)	C23—C24—C25—C26	0.5 (4)
O1—Tb1—N2—C12	74.93 (14)	C22—C21—C26—C25	0.4 (3)
O2—Tb1—N2—C12	127.46 (15)	C20—C21—C26—C25	177.4 (2)
N1—Tb1—N2—C12	5.57 (13)	C24—C25—C26—C21	−0.4 (4)
O4—Tb1—N2—C12	−101.64 (14)	Tb1—O6—C27—O5	2.0 (3)
C13—Tb1—N2—C12	101.65 (15)	Tb1—O6—C27—C28	−178.08 (14)
C20—Tb1—N2—C12	−88.66 (15)	Tb1^i^—O5—C27—O6	−20.6 (3)
Tb1^i^—Tb1—N2—C12	−135.30 (12)	Tb1^i^—O5—C27—C28	159.49 (13)
C2—N1—C1—C5	1.2 (3)	O6—C27—C28—C29	−145.65 (19)
Tb1—N1—C1—C5	−177.18 (14)	O5—C27—C28—C29	34.2 (3)
C2—N1—C1—C12	−178.29 (18)	O6—C27—C28—C33	30.1 (3)
Tb1—N1—C1—C12	3.3 (2)	O5—C27—C28—C33	−150.0 (2)
C1—N1—C2—C3	−0.4 (3)	C33—C28—C29—C30	−2.2 (3)
Tb1—N1—C2—C3	177.95 (16)	C27—C28—C29—C30	173.52 (19)
N1—C2—C3—C4	0.0 (3)	C28—C29—C30—C31	2.0 (3)
C2—C3—C4—C5	−0.4 (3)	C29—C30—C31—C32	−0.1 (4)
C3—C4—C5—C1	1.1 (3)	C30—C31—C32—C33	−1.6 (5)
C3—C4—C5—C6	−179.6 (2)	C31—C32—C33—C28	1.4 (4)
N1—C1—C5—C4	−1.6 (3)	C29—C28—C33—C32	0.5 (4)
C12—C1—C5—C4	177.89 (18)	C27—C28—C33—C32	−175.3 (2)
N1—C1—C5—C6	179.11 (19)	O7—C34—C35—C36	177.8 (3)
C12—C1—C5—C6	−1.4 (3)	O8—C34—C35—C36	−0.8 (3)
C4—C5—C6—C7	−178.0 (2)	O7—C34—C35—C40	−0.4 (4)
C1—C5—C6—C7	1.2 (3)	O8—C34—C35—C40	−179.0 (2)
C5—C6—C7—C8	−0.5 (3)	C40—C35—C36—C37	1.8 (4)
C6—C7—C8—C9	179.3 (2)	C34—C35—C36—C37	−176.4 (3)
C6—C7—C8—C12	0.1 (3)	C35—C36—C37—C38	−0.6 (5)
C12—C8—C9—C10	1.9 (3)	C36—C37—C38—C39	−0.8 (5)
C7—C8—C9—C10	−177.4 (2)	C37—C38—C39—C40	0.9 (5)
C8—C9—C10—C11	−0.9 (3)	C38—C39—C40—C35	0.3 (4)
C12—N2—C11—C10	2.0 (3)	C36—C35—C40—C39	−1.7 (4)
Tb1—N2—C11—C10	−173.94 (17)	C34—C35—C40—C39	176.5 (2)

Symmetry codes: (i) −*x*+2, −*y*, −*z*+2.

### Hydrogen-bond geometry (Å, °)

**Table:**

Cg1 is centroid of the C35–C40 phenyl ring.

**Table:**

*D*—H···*A*	*D*—H	H···*A*	*D*···*A*	*D*—H···*A*
O8—H1O8···O1	0.82	1.88	2.640 (3)	154
C2—H2A···O5^i^	0.93	2.40	3.036 (3)	125
C4—H4A···O2^ii^	0.93	2.45	3.172 (3)	135
C11—H11A···O6	0.93	2.40	2.969 (3)	120
C15—H15A···O7	0.93	2.58	3.448 (3)	155
C23—H23A···Cg1^iii^	0.93	2.57	3.462 (3)	160

Symmetry codes: (i) −*x*+2, −*y*, −*z*+2; (ii) *x*−1, *y*, *z*; (iii) −*x*+1, −*y*, −*z*+2.
